# Dysfunctional Postnatal Mitochondrial Energy Metabolism in a Patient with Neurodevelopmental Defects Caused by Intrauterine Growth Restriction Due to Idiopathic Placental Insufficiency

**DOI:** 10.3390/ijms25031386

**Published:** 2024-01-23

**Authors:** Martine Uittenbogaard, Andrea L. Gropman, Matthew T. Whitehead, Christine A. Brantner, Eliana Gropman, Anne Chiaramello

**Affiliations:** 1 Department of Anatomy and Cell Biology, School of Medicine and Health Sciences, George Washington University, 2300 I Street N.W., Washington, DC 20037, USA; mbogaard@gwu.edu (M.U.); eliana.gropman@gmail.com (E.G.); 2 Children’s National Medical Center, Division of Neurogenetics and Neurodevelopmental Pediatrics, Washington, DC 20010, USA; agropman@childrensnational.org; 3 Division on Neuroradiology, Department of Radiology, Children’s Hospital of Philadelphia, Philadelphia, PA 19104, USA; whiteheadm@chop.edu; 4 Electron Microscopy Core Imaging Facility, School of Dentistry and School of Medicine, University of Maryland Baltimore, Baltimore, MD 21201, USA; cbrantner@umaryland.edu

**Keywords:** placental insufficiency, fetal growth restriction, neurodevelopmental deficits, OXPHOS deficit, metabolic reprogramming, mitochondrial dysfunction

## Abstract

We report the case of a four-year-old male patient with a complex medical history born prematurely as the result of intrauterine growth restriction due to placental insufficiency. His clinical manifestations included severe neurodevelopmental deficits, global developmental delay, Pierre-Robin sequence, and intractable epilepsy with both generalized and focal features. The proband’s low levels of citrulline and lactic acidosis provoked by administration of Depakoke were evocative of a mitochondrial etiology. The proband’s genotype–phenotype correlation remained undefined in the absence of nuclear and mitochondrial pathogenic variants detected by deep sequencing of both genomes. However, live-cell mitochondrial metabolic investigations provided evidence of a deficient oxidative-phosphorylation pathway responsible for adenosine triphosphate (ATP) synthesis, leading to chronic energy crisis in the proband. In addition, our metabolic analysis revealed metabolic plasticity in favor of glycolysis for ATP synthesis. Our mitochondrial morphometric analysis by transmission electron microscopy confirmed the suspected mitochondrial etiology, as the proband’s mitochondria exhibited an immature morphology with poorly developed and rare cristae. Thus, our results support the concept that suboptimal levels of intrauterine oxygen and nutrients alter fetal mitochondrial metabolic reprogramming toward oxidative phosphorylation (OXPHOS) leading to a deficient postnatal mitochondrial energy metabolism. In conclusion, our collective studies shed light on the long-term postnatal mitochondrial pathophysiology caused by intrauterine growth restriction due to idiopathic placental insufficiency and its negative impact on the energy-demanding development of the fetal and postnatal brain.

## 1. Introduction

The American College of Obstetricians and Gynecologists defines intrauterine growth restriction (IUGR) as an estimated fetal weight of less than 10th percentile, which is a problem in about 10–15% of pregnant women [1,2]. This complex obstetric problem is commonly diagnosed antenatally. Its causes are multifactorial with consequences in fetal, neonatal, and adult life [3,4,5]. The most frequent causes of IUGR are maternal, placental, fetal, and/or genetic factors [6]. The maternal factors are diverse, such as age of the mother, interpregnancy interval, diabetes, hypertension, anemia, behavioral habits, and maternal infections [7]. Placental factors, such as weight of less 350 g, abnormal uteroplacental vasculature, placental mosaicism, and placental dysfunction and infections, result in a mismatch between the supply of nutrients by the placenta and the fetal demand for proper intrauterine growth [7]. Major congenital malformations, fetal infections, and metabolic disorders such as agenesis of the pancreas and fetal phenylketonuria are among the many fetal factors resulting in IUGR [8]. Finally, the genetic causes of IUGR have been reported to be polymorphisms on maternal, placental, or fetal genes [7]. For example, the homeobox genes, such as *DLX3*, *DLX4*, *ESL1*, *HLX1*, and *MSX2*, play an important role in the development and maintenance of the adult blood vasculature [9]. High concentrations of maternal endothelin-1 have been reported in IUGR pregnancies [10]. Genetic deletion of the fetal genes, *IGF1* and *SHOX*, has resulted in decreased fetal cell division and IUGR [11].

Fetal growth is essentially regulated by the interplay between maternal physiology and placental functions. The timing of inhibited fetal growth and its etiology dictate the type of growth abnormalities [8]. Symmetric IUGR constitutes 20–30% of the IUGR cases and results in an undersized fetus based on its timing during the first 24 gestational weeks when fetal growth occurs via hyperplasia to rapidly increase the number of cells without affecting the cellular size. It is mainly caused by chromosomic abnormalities including (but not limited to) trisomy 21, 13, and 18, or fetal infections, with toxoplasmosis and cytomegalovirus being the most frequent causes [12]. In contrast, asymmetric IUGR, which represents 70–80% of the IUGR cases, has a late onset during gestation during which cellular hypertrophy occurs. Thus, asymmetric IUGR leads to decreased fetal weight without affecting the total number of cells, but rather by reducing the overall cell size [13]. The reduced fetal growth rate results from a curtailed transfer of oxygen and nutrients between maternal and fetal blood due to an unfavorable intrauterine environment caused by placental insufficiency, maternal malnutrition, maternal smoking, maternal hypertension, uterine malformations, or chronic pulmonary disease [12,14].

IUGR impairs postnatal neurodevelopment and affects motor, cognitive, and behavioral functions [15,16,17,18]. IUGR infants display reduced brain volumes accompanied by delayed and disrupted myelination [19,20,21]. The placenta is a nutrient sensor and supplier that plays a pivotal role in providing metabolites required for mitochondrial metabolism to generate high levels of energy, which are essential for normal fetal growth and neural development [22]. Two recent studies have provided the first evidence of mitochondrial abnormalities in IUGR pregnancies, more specifically in terms of placental mitochondrial DNA copy numbers and mitochondrial variants [23,24]. Thus, we hypothesized that placental insufficiency mediated IUGR could provoke dysfunctional fetal mitochondrial metabolism, thereby resulting in deficient postnatal mitochondrial energy metabolism.

In this study, we tested our overall hypothesis in the case of a four-year-old male with a history of IUGR and suggested mitochondrial etiology based on lactic acidosis and low levels of citrulline provoked by the administration of Depakote. His complex medical history was characterized by severe neurodevelopmental deficits, global developmental delay, Pierre-Robin sequence, and intractable epilepsy with both generalized and focal features. In the absence of nuclear and mitochondrial pathogenic variants by deep sequencing of both genomes, his clinical and biochemical phenotypes remained without a unifying genetic diagnosis. However, live-cell functional metabolic investigations using patient-derived dermal fibroblasts provided evidence of a dysregulated OXPHOS pathway responsible for ATP synthesis, consistent with the suspected mitochondrial etiology of the proband’s phenotypic manifestations. Thus, our metabolic results validated the concept that sub-optimal levels of intrauterine oxygen and nutrients can alter fetal mitochondrial metabolic reprogramming toward OXPHOS, leading to deficient postnatal mitochondrial energy metabolism.

## 2. Results 

### 2.1. Clinical History

**Figure 1:**
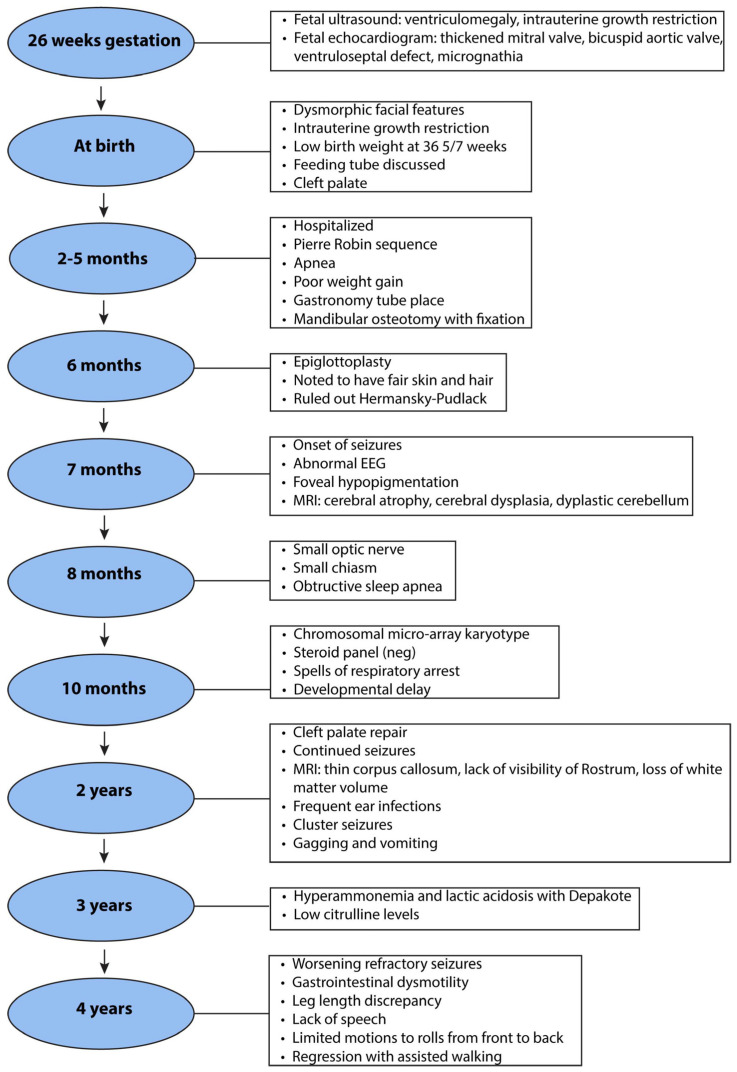
Highlights of the proband’s clinical history.

The four-year-old male proband was born at 36 weeks gestational age with a history of intrauterine growth restriction (IUGR) and a complex medical history of Pierre-Robin sequence (PRS) with the classical triad of micrognathia, glossoptosis with severe obstructive sleep apnea, and cleft palate, as well as of microcephaly, chronic static encephalopathy, global developmental delay, congenital hypotonia, and intractable epilepsy with both generalized and focal features, suggestive of a mitochondrial etiology without a unifying genetic diagnosis (Figure 1). 

**Figure 2:**
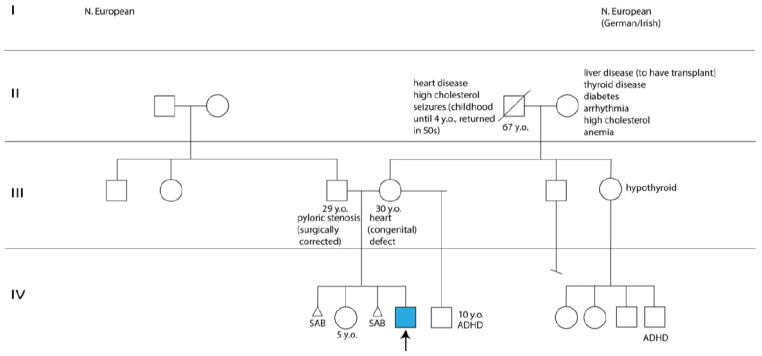
Pedigree tree with the proband identified by an arrow and a blue square. Abbreviations: ADHD, attention deficit and hyperactivity disorder; SAB, spontaneous abortion; y.o., years old.

The proband’s parents, a 30-year-old mother with a congenital heart defect and a healthy 29-year-old father, were of northern European descent and nonconsanguineous. The mother had a healthy 5-year-old daughter with the proband’s father, and a 10-year-old son with a separate partner, diagnosed with attention-deficit/hyperactivity disorder. Worth noting was the mother’s two pregnancy losses before and after her daughter’s birth, one of which required a dilation and curettage procedure (Figure 2). The mother was a light smoker, who took prenatal vitamins and did not experience common maternal health conditions, such as diabetes, pre-eclampsia, thyroid problems, infection, rash, fever, hypertension, or bleeding during pregnancy. Moreover, the family history was noncontributory for the proband’s complex phenotypic manifestations (Figure 2).

**Figure 3:**
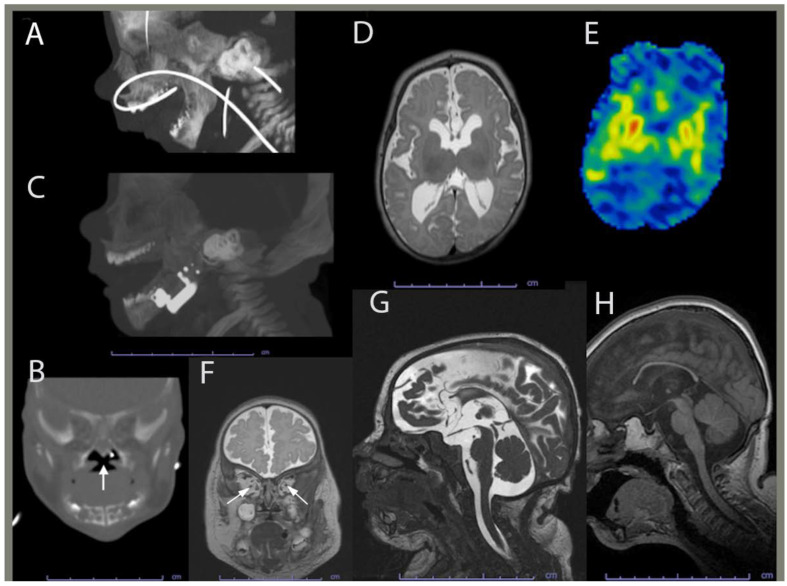
Computed tomography and brain magnetic resonance imaging (MRI) of the proband. (**A**) Maxillofacial computed tomography (CT) in sagittal planes performed at 35 days shows mandibular hypoplasia. (**B**) Maxillofacial CT in coronal planes performed at 35 days shows a U-shaped cleft palate with the white arrow indicating components of Pierre-Robin sequence. (**C**) Sagittal image from a postoperative face CT at 3 months shows improved mandibular and maxillary proportions. (**D**) Brain MRI axial T2WI image performed at 4 months shows myelination-related signal hypointensity being mildly deficient consistent with hypomyelination or delayed myelination. (**E**) Brain MRI axial ASL perfusion image showing the basal ganglia being hyperperfused (orange and red hues). (**F**) Brain MRI coronal T2WI indicating that the optic nerves are thin, consistent with volume loss and/or hypoplasia (arrows). (**G**) Brain MRI sagittal midline FIESTA image showing moderate diffuse cerebral gray and white matter and mild brainstem volume loss and/or hypoplasia with commissural thinning, decreased white-matter depth, and ex vacuo ventriculomegaly. The corpus callosum is hypogenetic/dysgenetic and hypoplastic, being diffusely thin with absence of the rostrum. (**H**) Brain MRI at 4 years, showing relatively similar cerebral and brainstem volume loss and/or hypoplasia.

During pregnancy, the mother did not gain much gestational weight and reported decreased fetal movements. Subsequently, she was monitored by a maternal–fetal-medicine specialist. The Panorama serum screen was normal. At 15 weeks, a fetal echocardiogram, which was prompted by the detection of ventricular enlargement by level II ultrasound, revealed a thickened mitral valve, a bicuspid aortic valve, and a small muscular ventricular septal defect. Amniocentesis and chorionic villus sampling were declined. At 26 weeks, a fetal ultrasound revealed ventriculomegaly. At 35 weeks, a postnatal head ultrasound confirmed supratentorial ventriculomegaly along with decreased cerebral white-matter volume in keeping with white-matter volume loss and/or hypoplasia. A same-day computed tomography (CT) showed mandibular hypoplasia and a U-shaped cleft palate, both components of PRS (Figure 3A,B). At 36-5/7 weeks, vaginal delivery was induced due to IUGR and fetal growth arrest. 

At birth, the proband weighed 3 lbs and 2 oz and was admitted to the newborn intensive care unit for IUGR and feeding difficulties. The proband’s dysmorphic facial features led to the diagnosis of PRS with the clinical triad of airway obstruction, glossoptosis with cleft palate, and micrognathia. He required phototherapy for jaundice and passed the newborn hearing screen before being discharged 21 days later. Between the age of two and five months, the proband was hospitalized due to poor weight gain and required placing of a gastrotomy tube, and a mandibular osteotomy with fixation. Mandibular morphology was more normally proportioned on CT following surgical distraction (Figure 3C). Brain magnetic resonance imaging (MRI) at four months revealed corpus callosum dysgenesis, a deficient myelination-related signal, basal ganglia hyperfusion, optic pathway volume loss and/or hypoplasia, and brain volume loss and/or hypoplasia (Figure 3D–G). He was then referred to our ophthalmology clinic to evaluate the reduced optic pathway volume; he could blink to light but could not fix or follow it.

At six months old, the proband underwent an epiglottoplasty to alleviate severe apnea. He also showed hypopigmentation of the skin, hair, and fovea. However, the diagnosis of the Hermansky–Pudlak syndrome was ruled out by a normal analysis of the multigene panel that included the *AP3B1*, *AP3D1*, *BLOC1S3*, *BLOC1S5*, *BLOCS1S6*, *DTNBP1*, *HPS1*, *HPS3*, *HPS4*, *HPS5*, and *HPS6* genes. The proband’s karyotype was also normal. Thus, the proband was diagnosed with unspecified oculocutaneous albinism. At the age of seven months, the proband had an onset of seizures with abnormal electroencephalogram. He exhibited failure to thrive and developmental delays with a developmental level estimated to be that of a two-to-three-month-old infant. The thyroid screen and sterol panels were normal.

At the age of 15 months, the proband’s brain MRI showed no evidence of an acute intracranial disease process but revealed diffuse moderate cerebral gray and white matter with volume loss, mild brainstem hypoplasia, deficient myelination, diffuse thinning of the corpus callosum, and a lack of visibility of the rostrum, consistent with corpus callosum dysgenesis. These findings could have reflected loss of white-matter volume and superimposed mild corpus callosum dysgenesis, all suggestive of delayed myelination or hypomyelination. 

At the age of 20 months, a brain CT scan showed further thinning of the corpus callosum and stable diffuse decrease in the cerebral volume. At the age of 27 months, the proband’s brain MRI indicated no significant changes in his moderate volume loss of cerebral white matter and brainstem but showed a slight increase in the associated ex vacuo ventriculomegaly in proportion to his head growth. At four years of age, an MRI revealed a relatively similar brain volume reduction when considering interval patient growth and development (Figure 3H). Although myelination progressed, it remained deficient for the proband’s age, and therefore consistent with hypomyelination. 

Given the proband’s poor postnatal growth, microcephaly, static encephalopathy, focal and generalized epilepsy, and brain abnormalities without a unifying genetic diagnosis, pituitary abnormalities and growth hormone deficiency were explored. His IGF-1, IGFBP-3, and morning cortisol levels were normal. At the age of three, he was prescribed Depakote for seizures, which triggered hyperammonemia and lactic acidosis. He was switched to Keppra, clonazepam, and clonidine. When seizures lasted more than five minutes or had a frequency of three in one hour, the patient was administered Diastat and immediately hospitalized. At the age of four, the proband had worsening refractory seizures and gastrointestinal dysmotility, and acquired unequal leg length and global developmental delay with absence of speech, movements limited to rolling from front to back, and regression of assisted walking with no weight bearing on the left.

Collectively, the proband’s symptoms of hypotonia, global developmental delays, intractable epilepsy with both generalized and focal features, static encephalopathy, reaction to Depakote, and low levels of citrulline, led to a suspected mitochondrial etiology. However, the dual genome panel was uninformative in the absence of any mitochondrial pathogenic variants or large deletion of the mitochondrial genome. His dual genome panel only revealed a single heterozygous variant of uncertain significance, c.712C > T (p.R238C), mapping in the *ADCK3* gene involved in the coenzyme Q synthesis pathway. Whole exome sequencing of the nuclear genome and LR-PCR–MPS of the mitochondrial genome failed to reveal any nuclear or mitochondrial pathogenic variants.

### 2.2. Functional Studies of the ATP Metabolism

**Figure 4:**
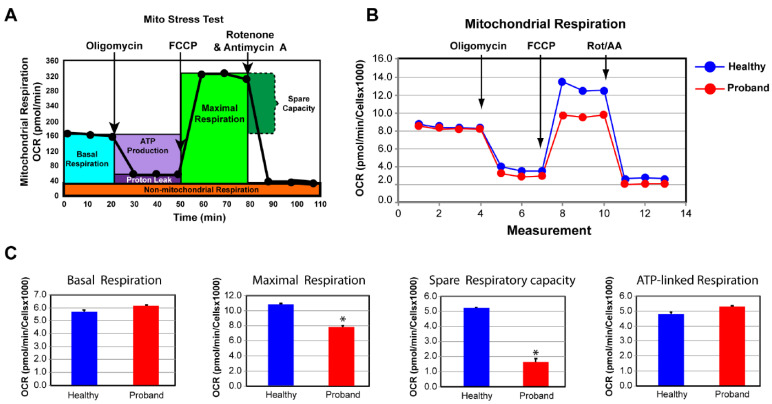
The proband’s fibroblasts exhibited an impaired bioenergetic capacity. (**A**) Profile of the oxygen consumption rate (OCR) adapted from Agilent Technologies brochure of the Mitochondrial Stress Test. (**B**) Comparison of OCR responses of the proband and a healthy subject. (**C**) Quantitative basal respiration, ATP-linked respiration, maximal respiration, and spare respiratory capacity data of the proband and the healthy subject. Data are represented as means ± S.E.M., *n* = 3 of three independent experiments. * indicates statistically significant differences with a *p* value of ≤0.005 between the proband and the healthy subject.

To investigate whether an underlying mitochondrial etiology could be contributing to the patient’s symptoms for a neurodevelopmental disorder of an unspecified genetic diagnosis, we performed a skin biopsy on the four-year-old proband. As a control subject, we used commercially available dermal fibroblasts from a healthy 10-year-old subject whose metabolic profile was already characterized and comparable to two other healthy subjects [25]. By means of oxygen consumption rate (OCR) as a functional indicator of the mitochondrial energy metabolism, we assessed OXPHOS parameters using the Mitochondrial Stress Test assay (Figure 4). We found that only the basal respiration and its related parameter, ATP-linked respiration, remained unchanged, when compared to those of a healthy subject (Figure 4C). In contrast, the maximal respiration capacity evoked by exposure to the protonophore fluoro 3-carbonyl cyanide-methoxyphenyl hydrazine (FCCP) decreased by 28% (Figure 4C). More critical was the 69% decline in the spare respiratory capacity, essential for the proband’s cells to avert an ATP crisis upon energy demand (Figure 4C). Thus, the proband’s fibroblasts exhibited a dysregulated OXPHOS pathway congruent with his severe neurological symptoms.

**Figure 5:**
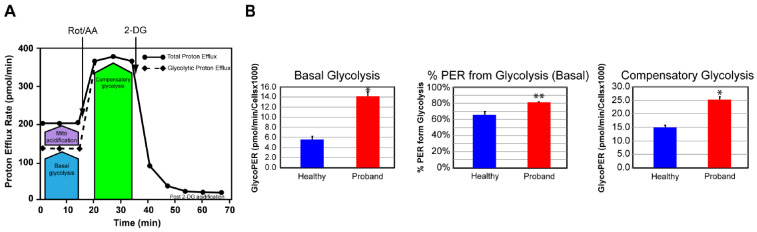
The proband’s fibroblasts displayed an increased proton efflux rate indicative of a stimulated glycolysis pathway. (**A**) Schematic representation of proton efflux adapted from the Agilent Technologies brochure of the Glycolytic Rate Test. (**B**) Quantitative data of basal glycolysis, compensatory glycolysis, and mitochondrial acidification. Data are represented as means ± S.E.M., *n* = 3 of three independent experiments. * and ** indicate statistically significant differences with a p value of 0.0001 and 0.0037 between the proband and the healthy subject.

We next examined the glycolytic metabolism of the proband’s fibroblasts to determine whether they could switch to glycolysis for increased ATP synthesis. To this end, we used the glycolysis rate assay to accurately assess the glycolytic activity by correlating one-to-one with lactate accumulation. The total proton efflux rate (PER) and the glycolytic proton efflux rate (GlycoPER) were measured using both OCR and ECAR values to account for mitochondrial (CO_2_) acidification from the mitochondrial TCA cycle (Figure 5A) [26]. We found that the proband’s fibroblasts showed a 180% increase in basal glycolysis, which was confirmed by an increase in PER to 81%, compared with 66% in dermal fibroblasts from the healthy subject (Figure 5B). We next measured the compensatory glycolysis response as an indicator of metabolic reprogramming toward glycolysis subsequent to an OXPHOS crisis provoked by exposure to rotenone and antimycin A. We noticed that the proband’s fibroblasts were endowed with a strong compensatory glycolysis response that was 40% superior to that of healthy fibroblasts (Figure 5B).

**Figure 6:**
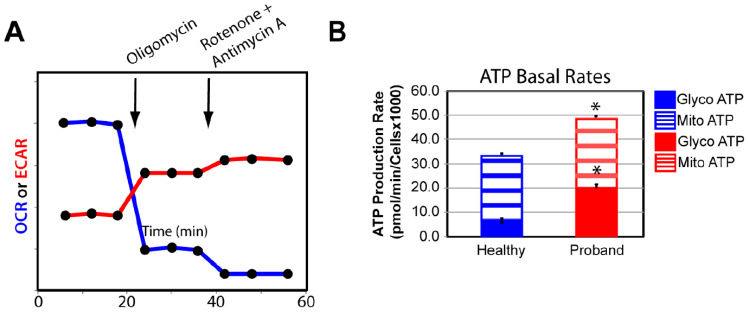
The proband’s fibroblasts showed a disturbed energy metabolic phenotype. (**A**) Schematic representation of the Agilent Seahorse XFp Real-Time ATP rate assay. Both oxygen consumption rate (OCR) and extracellular acidification rate (ECAR) of live fibroblasts from the proband and a healthy subject were simultaneously measured upon injection of the mitochondrial inhibitor oligomycin followed by a mixture of rotenone and antimycin A. (**B**) Quantification of the basal rate of ATP production from glycolysis (blue for the healthy subject and red for the proband) and mitochondrial oxidative phosphorylation (OXPHOS) (blue hatched column for the healthy subject and red hatched column for the proband). Data are represented as means ± S.E.M., *n* = 3 of three independent experiments. * indicates statistically significant differences with a *p* value of 0.0002 between the proband and the healthy subject.

We confirmed our OXPHOS and glycolytic results by measuring the basal rate of ATP production from glycolysis and the OXPHOS pathway using the XFp Real-Time ATP Rate Assay Kit. Both OCR and extracellular acidification rate (ECAR) were simultaneously measured upon injection of oligomycin followed by injection of rotenone and antimycin A to fully inhibit mitochondrial ATP production (Figure 6A). We detected similar basal rates of ATP production in the proband’s fibroblasts and healthy fibroblasts, corroborating our finding of comparable basal respiration from the OXPHOS pathway between the diseased and healthy fibroblasts (Figure 6B). Our results revealed a 233% increase in the rate of ATP production from basal glycolysis, validating our previous findings of metabolic switch from OXPHOS to glycolysis in the proband’s fibroblasts to increase ATP synthesis (Figure 6B).

### 2.3. Mitochondrial Morphometric Examination of the Proband’s Fibroblasts

**Figure 7:**
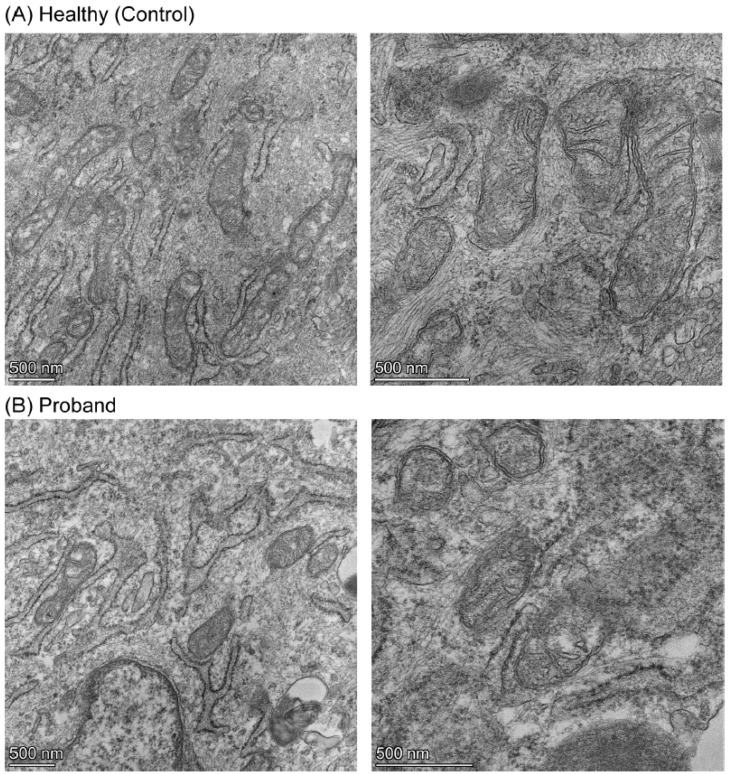
Mitochondrial morphometric analysis illustrated the immature morphology and poorly developed cristae of the proband’s mitochondria. (**A**) Mitochondria from a healthy subject at low and high magnifications on the left and right panels, respectively. (**B**) Mitochondria from the proband at low and high magnifications on the left and right panels, respectively. The scale bar (500 nm) is indicated at the bottom left corner of each micrograph.

We then performed a mitochondrial morphometric analysis using transmission electron microscopy to investigate the proband’s mitochondrial ultrastructure. The proband’s fibroblasts harbored fewer mitochondria and these were of smaller size than those in healthy fibroblasts (Figure 7). More specifically, the proband’s mitochondria exhibited small and rare cristae, similar to those observed in immature mitochondria [27], while mitochondria from the healthy subject had elongated mitochondria with normal cristae (Figure 7). These mitochondrial morphometric results confirmed a deficiency in mitochondrial energy metabolism and a reliance on glycolysis for ATP synthesis (Figure 4, Figure 5 and Figure 6).

## 3. Discussion

Our results generated with fibroblasts from a four-year-old male born prematurely due to intrauterine growth restriction (IUGR) corroborated our hypothesis that sub-optimal levels of intrauterine oxygen and nutrients can alter fetal mitochondrial metabolic reprogramming toward OXPHOS, leading to deficient postnatal mitochondrial energy metabolism.

The proband had a complex medical history with profound neurodevelopmental and global defects, microcephaly, congenital hypotonia, intractable epilepsy with both generalized and focal features, and the dysmorphic syndrome Pierre-Robin sequence (PRS) with the classical clinical triad of micrognathia, glossoptosis with severe obstructive sleep apnea, and cleft palate. The absence of chromosomal abnormalities and genetic factors known to cause PRS, such as the critical chondrogenic regulator SOX9, supported the extrinsic abnormalities and neurological/neuromuscular abnormalities of the proband as potentially being caused by embryonic pathogenic events [28]. Both were consistent with the proband’s congenital hypotonia and IUGR, evocative of an in-utero compression of his mandible. Our live-cell mitochondrial metabolic analyses validated the patient’s suspected mitochondrial etiology based on his low levels of citrulline and lactic acidosis elicited by Depakote. Moreover, our mitochondrial morphometric analysis revealed an immature mitochondrial morphology consistent with the patient’s deficient oxidative phosphorylation (OXPHOS) and neurodevelopmental deficits.

The genetic causes of the proband’s complex phenotypic manifestations remained enigmatic in the absence of nuclear and/or mitochondrial pathogenic variants by whole exome sequencing of the nuclear genome and deep sequencing of the mitochondrial genome. Thus, the most probable cause was uteroplacental insufficiency and IUGR. Placental insufficiency triggers inadequate supply of substrates such as glucose, fatty acids, amino acids, and oxygen, which impairs the fetal energy metabolism and, consequently, fetal growth and development [29,30,31]. IUGR is commonly associated with reduced blood flow through the placenta, combined with limited invasion of the decidua and maternal blood vessels [32] [. This leads to curtailed levels of oxygen and fuel substrates resulting in fetal mitochondrial dysfunction and the proband’s grey-matter and white-matter pathologies revealed by brain magnetic resonance imaging (MRI), such as hypomyelination, corpus callosum dysgenesis, and decreased cerebral volume. Since mitochondrial ATP synthesis is coupled with oxygen consumption by Complex IV, low fetal levels of oxygen most likely decrease the overall OXPHOS activities to generate antenatal chronic energy deficit. It is well documented that the developing brain requires high levels of energy [33,34]. This infers that those deficits in brain structure and functions are the consequence of antenatal and postnatal alteration of the mitochondrial energy metabolism. 

Most notable is the emerging evidence that maternal and fetal stressors induce dysregulation of the placental epigenome, such as histone post-translational modifications, thereby resulting in IUGR and postnatal neurological disorders [35]. This is congruent with our findings that epigenetic regulation via post-translational modifications of histones calibrates the mitochondrial OXPHOS metabolism to match the high energy demand required during neuronal differentiation [36]. Thus, we cannot exclude that dysregulation of the placental epigenome could, in part, result in the proband’s dysregulated mitochondrial OXPHOS metabolism and severe postnatal neurodevelopmental defects. Furthermore, this is consistent with the fact that whole exome sequencing (WES) and deep sequencing of the nuclear and mitochondrial genomes failed to reveal any nuclear or mitochondrial pathogenic variant, respectively.

Congruent with the patient’s brain MRI was the severe deficit in the spare respiratory capacity revealed by our live-cell functional mitochondrial OXPHOS investigations. Since firing neurons require 80% of the spare respiratory capacity for assuming synaptic activities [37], the proband’s developing brain did not have a sufficient reserve of energy to avert an ATP crisis upon such high energy demand. Congruent with our mitochondrial functional investigations was our mitochondrial morphometric analysis showing the proband’s immature mitochondrial morphology with poorly developed cristae, a morphological indicator of limited OXPHOS capacity and mitochondrial ATP synthesis [25,27,38]. In keeping with the proband’s abnormal mitochondrial morphology and deficit of the OXPHOS pathway was the proband’s metabolic adaptation to favor glycolysis for ATP synthesis, demonstrated by an increased basal glycolytic response and compensatory glycolytic response as an attempt to overcome the OXPHOS-based energy deficit. 

Interestingly, the immature mitochondrial morphology, the mitochondrial energy deficit, and the metabolic adaptive response toward glycolysis are hallmarks of mitochondria from undifferentiated human embryonic stem cells during the early stages of mammalian embryonic development [39]. This mitochondrial metabolic profile is programmed to support proliferation during the pre-implantation embryonic development and early fetal developmental stages [40]. More specifically, early fetal developmental stages are characterized by low levels of OXPHOS and oxygen consumption, combined with high glycolysis via passive diffusion of glucose through the placenta [41]. At later fetal developmental stages, mitochondria undergo functional metabolic adaptation toward the OXPHOS metabolism to meet the high energy requirement for cell specification and differentiation [42]. Such mitochondrial metabolic flexibility is necessary for proper fetal growth and development throughout the different in-utero stages [43,44]. Various animal models for IUGR support such metabolic plasticity toward glycolysis with an early activation of hepatic glucose production as a substrate for ATP synthesis, a reactive process that is absent in normal fetuses [45,46].

In conclusion, our collective studies shed light on the long-term postnatal mitochondrial pathophysiology caused by intrauterine growth restriction due to placental insufficiency and its negative impact on the energy-demanding development of the fetal and postnatal brain.

## 4. Materials and Methods

### 4.1. Editorial Policies and Ethical Considerations

This study was approved by the Institutional Review Board of the George Washington University and Children’s National Medical Center and was conducted in accordance with the ethical principles of the Declaration of Helsinki of 1975 (revised 1983). Patient skin biopsy was performed after receiving written informed consent with permission to study the derived dermal fibroblasts.

### 4.2. Skin Biopsy and Fibroblast Culture

A 3 mm skin biopsy was performed on a 4-year-old male proband, from which dermal fibroblasts were derived in Dulbecco’s Modified Eagle Medium (DMEM; Gibco, Waltham, MA, USA) supplemented with 2 mM glutamine, 2.5 mM pyruvate, 0.2 mM uridine, FGF-2 (10 ng/mL), and 20% fetal bovine serum, as described in [47]. Derived dermal fibroblasts were frozen at passage 2 and never used beyond passage 10. Human primary dermal fibroblasts from a healthy 10-year-old male (Cat# GM03377E) were obtained from the Coriell Cell Repositories (Camden, NJ, USA).

### 4.3. Clinical Genetic Diagnosis

Whole exome sequencing (WES) and long-range PCR followed by massively parallel sequencing (LR-PCR–MPS) of the mitochondrial genome were performed by GeneDx (Gaithersburg, MD, USA) using dermal fibroblasts derived from the proband’s skin biopsy.

### 4.4. Transmission Electron Microscopy

Dermal fibroblasts of the proband and a healthy subject (control) were fixed in 2.5% glutaraldehyde (Electron Microscopy Sciences, Hatfield, PA, USA) and 1% paraformaldehyde in 0.12 M sodium cacodylate buffer (Electron Microscopy Sciences) for 20 min at room temperature followed by 40 min on ice, as described in [48]. Samples were imaged with an FEI Talos F200X transmission electron microscope (ThermoFisher, Waltham, MA, USA).

### 4.5. Live-Cell Measurements of Mitochondrial Respiratory and Glycolytic Activity

The bioenergetic status of dermal fibroblasts derived from the patient and the healthy control subject was measured using the Seahorse Extracellular Flux XFp Analyzer (Agilent Technologies; Santa Clara, CA, USA), as described in [47]. Optimal cell density (5000/well) and the uncoupler FCCP (fluoro 3-carbonyl cyanide-methoxyphenyl hydrazine; 2 µM) were determined using the Cell Energy Phenotype Test Kit. Dermal fibroblasts were seeded in triplicate on poly-D lysine-coated plates and incubated for 24 h at 37 °C in 5% CO_2_ atmosphere. Prior to the assay, the supplemented DMEM medium was changed to unbuffered base medium supplemented with 2 mM glutamine (Invitrogen, Waltham, MA, USA), 2 mM pyruvate (Sigma; St Louis, MO, USA), and 7.1 mM glucose (Sigma) and adjusted to pH 7.4 with NaOH. Using the XFp Mito Stress Test Kit, oxygen consumption rate (OCR) and extracellular acidification rate (ECAR) were measured under basal conditions and after sequential injections of oligomycin (1 µM), FCCP (2 µM), and a mix of rotenone and antimycin A (1 µM), following the manufacturer’s recommendations. Quantification analyses of key functional parameters for mitochondrial respiration via OXPHOS were carried out using the Agilent XF Mito Stress Test Report Generator. OCR was normalized to the number of cells quantified after the conclusion of the assay using the Cytation 1 equipment (Bio Tek, Winooski, VT, USA) and expressed in pmol/min/cell X 1000.

Using the Seahorse XFp Real-Time ATP Rate Assay Kit, we simultaneously quantified the rate of ATP produced by the two main bioenergetic pathways, OXPHOS and glycolysis, to calculate the total cellular rate of ATP production according to the manufacturer’s recommendations. Prior to the assay, the culture medium was switched to the phenol-red and bicarbonate-free medium assay containing 5 mM HEPES, 2 mM glutamine (Invitrogen), 2 mM pyruvate (Sigma), and 7.1 mM glucose (Sigma), and adjusted to pH 7.4 with NaOH. OCR and ECAR were measured under basal conditions and after sequential injections of oligomycin (1.5 µM) and a mix of rotenone and antimycin A (0.5 µM). 

Using the XFp Glycolytic Rate Assay Kit, we analyzed the glycolytic rate by quantifying the total proton efflux and the glycolytic proton efflux, as described in [48]. Prior to the assay, the supplemented DMEM medium was changed to the XF base medium without phenol red but supplemented with 2 mM glutamine, 10 mM glucose, 1 mM pyruvate, and 5.0 mM HEPES. OCR and ECAR were measured under basal conditions and after sequential injections of rotenone/antimycin A (0.5 µM) and 2-DG (50 mM). 

All the data from three independent experiments, each including three technical replicates, were normalized to cell numbers after the assay and plotted as OCR (pmol/min/cell ± S.E.M.) and ECAR (mpH/min/cell ± S.E.M.), as a function of time using the Agilent Seahorse MultiReport Generator software (Wave pro 10.1.0). Statistical analyses were performed using the unpaired Student’s *t*-test with a *p* value of less than 0.05 considered statistically significant.

## Data Availability

Data is containing within the article.

## Abbreviations

COXPD = combined oxidative phosphorylation deficiency; CT = computed tomography; DMEM = Dulbecco’s Modified Eagle Medium; ECAR = extracellular acidification rate; EEG = electroencephalogram; FCCP = fluoro 3-carbonyl cyanide-methoxyphenyl hydrazine; FGF-2 = fibroblast growth factor-2; FIESTA = fast imaging employing steady-state acquisition; GlycoPER = glycolytic proton efflux rate; HEPES = 4-(2-hydroxyethyl)-1-piperazineethanesulfonic acid; IUGR = intrauterine growth restriction; LR-PCR–MPS = long-range PCR followed by massively parallel sequencing; MRI = magnetic resonance imaging; NGS = next-generation sequencing; PER = proton exchange rate; PRS = Pierre-Robin sequence; OCR = oxygen consumption rate; OXPHOS = oxidative phosphorylation; T2WI = T2-weighted image; WES = whole exome sequencing.
